# Comparison of activity indexes for recognizing enzyme mutants of higher activity with uricase as model

**DOI:** 10.1186/1752-153X-7-69

**Published:** 2013-04-17

**Authors:** Juan Feng, Hongbo Liu, Xiaolan Yang, Ang Gao, Juan Liao, Liping Feng, Jun Pu, Yanling Xie, Gaobo Long, Yuanli Li, Fei Liao

**Affiliations:** 1 Unit for Analytical Probes and Protein Biotechnology, Key Laboratory of Clinical Laboratory Diagnostics of the Education Ministry, College of Laboratory Medicine, Chongqing Medical University, Chongqing, 400016, China

**Keywords:** Activity concentration, Interference, Positive candidate, Specific activity, Spectrometric methods, Threshold, Uricase

## Abstract

**Background:**

For screening a library of enzyme mutants, an efficient and cost-effective method for reliable assay of enzyme activity and a decision method for safe recognition of mutants of higher activity are needed. The comparison of activity concentrations of mutants in lysates of transformed *Escherichia* coli cells against a threshold is unsafe to recognize mutants of higher activity due to variations of both expression levels of mutant proteins and lysis efficiency of transformed cells. Hence, by a spectrophotometric method after verification to measure uricase activity, specific activity calculated from the level of total proteins in a lysate was tested for recognizing a mutant of higher activity.

**Results:**

During uricase reaction, the intermediate 5-hydroxyisourate interferes with the assay of uric acid absorbance, but the measurement of absorbance at 293 nm in alkaline borate buffer was reliable for measuring uricase initial rates within a reasonable range. The level of total proteins in a lysate was determined by the Bradford assay. Polyacrylamide gel electrophoresis analysis supported different relative abundance of uricase mutant proteins in their lysates; activity concentrations of uricase in such lysates positively correlated with levels of total proteins. Receiver-operation-curve analysis of activity concentration or specific activity yielded area-under-the-curve close to 1.00 for recognizing a mutant with > 200% improvement of activity. For a mutant with just about 80% improvement of activity, receiver-operation-curve analysis of specific activity gave area-under-the-curve close to 1.00 while the analysis of activity concentration gave smaller area-under-the-curve. With the mean plus 1.4-fold of the standard deviation of specific activity of a starting material as the threshold, uricase mutants whose activities were improved by more than 80% were recognized with higher sensitivity and specificity.

**Conclusion:**

Specific activity calculated from the level of total proteins is a favorable index for recognizing an enzyme mutant with small improvement of activity.

## Background

Evolution biotechnology is powerful to discover new enzymes in chemical biology [1-4]. The practice of evolution biotechnology requires an efficient and cost-effective analytical method for reliable assay of catalytic capacities of mutants, and a suitable decision method for recognizing mutants of higher catalytic capacity as positive candidates or potential hits. In general, there should be mutagenesis on multiple sites of an enzyme to significantly improve the catalytic capacity; random mutagenesis of all possible sites of an enzyme will produce a huge library to burden the screening process. Hence, the design of focused libraries of mutants of an enzyme via mutagenesis of just a few sites is favorable, but a suitable decision method is required to recognize positive candidates with small improvement of catalytic capacity [5-8].

Catalytic capacity of an enzyme is measurable only with its sample of the highest purity. To practice the screening of a library, however, crude enzymes of mutants in lysates of transformed *Escherichia* coli cells after induced expression have to be employed as samples to measure activities for comparison against a suitable threshold derived from that of the starting material [6-13]. In general, the concentration of an enzyme in a lysate reflected by its activity (denoted as activity concentration hereafter) or specific activity can be utilized as activity index for comparison. The comparison of activity concentration of a mutant in its lysate against a threshold to recognize positive candidates is utilized in classical decision methods, but the variations in levels of mutant proteins due to differences in the inoculation quantity of a clone, amplification efficiency of the clone, induced expression levels and/or lysis efficiency of transformed cells reduce the safety for recognizing positive candidates. The comparison of specific activity may facilitate recognizing a mutant of small improvement of activity, but is prevented from practicing by the lack of efficient methods for selective assay of a mutant protein in its lysate. Alternatively, the quantification of total proteins in a lysate by a conventional protein assay may yield a practical specific activity to facilitate recognizing a positive candidate, but the effectiveness of this approach has not been reported yet.

Uricases are biodrugs to treat refractory gout [14-17], and pivotal analytical tools to measure serum uric acid [18]. Any application of uricase requires its catalytic capacity as high as possible [19-21], but available wildtype uricases all have low catalytic capacity. To date, uricase catalytic mechanisms are unknown and evolution biotechnology is practical to obtain uricase mutants of higher catalytic capacity. Uricase catalyzes the oxidation of uric acid into hydrogen peroxide and 5-hydroxyisourate (HIU) that spontaneously decomposes into allantoin of no absorbance at wavelengths over 280 nm [22]. Peroxidase-coupled and ferrous/ferric ion-mediated assays of hydrogen peroxide can measure uricase activity by quantifying absorbance or fluorescence [23-27], but require the termination of uricase reaction and thus reduce efficiency for screening uricase mutants. Notably, there must be a large portion of uric acid leftover in reaction solution to measure uricase initial rates; residual uric acid in uricase reaction solutions usually interferes with such methods for quantifying hydrogen peroxide [28,29]. On the other hand, uric acid has a strong absorbance peak at 293 nm and the assay of uric acid absorbance has the highest efficiency and the lowest cost for screening uricase mutants. However, HIU has an absorption peak around 302 nm and may interfere with the assay of uricase initial rates by measuring uric acid absorbance [30,31]. Therefore, the screening of uricase mutants needs a decision method to safely recognize positive candidates and the verification of the assay of uric acid absorbance for measuring uricase activity.

The uricase of *Bacillus fastidious* is a promising biodrug for treating refractory gout and a suitable tool for serum uric acid assay [32-34]; we cloned this uricase gene and obtained some mutants with small differences in catalytic capacities. In this report, the assay of uric acid absorbance at 293 nm was verified for measuring uricase activity; specific activity calculated from the level of total proteins in a lysate was tested as the index for comparison against a threshold for recognizing a positive candidate.

## Materials and methods

### Reagents, chemicals and apparatus

Uric acid was from Sigma-Aldrich. Boric acid, tri-(hydroxylmethyl)-aminomethane (Tris), and sodium borate were common reagents of analytical grade or better. Water was re-stilled before use. *p*ET28a vector carrying on the coding sequence for the wildtype intracellular uricase from *Bacillus* fastidious A.T.C.C. 29604 was that we used previously (gene id FJ393559, protein id ACR09749.1) [33]. By site-directed mutagenesis or the substitution of a fragment with an intended sequence, uricase mutants with different sequences of the N-terminus and/or mutagensis of the unique cysteine residue into aspartic acid residue were generated (Additional file 1: sheet 1, mutant sequences and summary; these vectors were constructed via technical service provided by Taihe Biotechnolyg Co. Ltd, Beijing 100070, China). *Escherichia* coli cell strain BL21 (DE3) as the host to harvest vectors of such uricases for induced expression was provided by Sangon Biotechnology (Shanghai, China). DEAE-cellulose was from Whatman (Kent, UK). Xinmao UV 7504 spectrophotometer (http://www.china-xinmao.com) was used throughout the work.

### Recombinant expression and purification of the wildtype uricase

The expression of the wildtype uricase followed that described previously [33]. After the induced expression with isopropyl-β-*D*-thiogalactoside (IPTG) for 18 h at 16°C, *Escherichia* coli cells BL21 (DE3) were harvested, and lysed by sonication; the soluble uricase was purified over two consecutive DEAE-cellulose chromatography *via* the elution with 0.10 M Tris–HCl at pH 8.0 plus a linear gradient of NaCl from 0 to 0.40 M. Fractions with the activities over 6.0 U·mg^-1^ protein were pooled, and dialyzed against 50.0 mM sodium borate buffer (Na_2_B_4_O_7_·10H_2_O) at pH 9.2 for 24 h at 4°C with several changes of the buffer, and then stored at 4°C before use. Uricase activities were calibrated at 25°C with 0.075 mM uric acid in borate buffer at pH 9.2 by measuring absorbance at 293 nm. One unit of uricase oxidized one micromole uric acid per min calculated with the extinction coefficient of 11.5 (mM) ^-1^·cm^-1^[33,35].

### Monitor of uricase reaction and estimation of Michaelis-Menten constant

Borate buffer was prepared with a required ratio of 50.0 mM sodium borate (Na_2_B_4_O_7_·10H_2_O) to 0.20 M boric acid [35]. Each reaction mixture in a total of 0.80 mL contained 50 μL uricase solution and 0.75 ml solution of uric acid in the borate buffer at an indicated pH. The solutions of uric acid were pre-incubated at (25 ± 0.5)°C for 20 min before use and prepared every day. Reaction was initiated by the addition of uricase solution; absorbance was recorded in an isolated small room air-conditioned at 25°C, after a lag time of 20 s at proper intervals within 5 min, unless otherwise stated [36,37]. The wildtype intracellular uricase of *Bacillus* fastidious A.T.C.C. 29604 has Michaelis-Menten constant (*K*_m_) over 0.20 mM and uric acid over 0.40 mM is needed for estimating *K*_m_[30,31]; the uricase of Asahi-Kasei (http://www.asahi-kasei.co.jp/shindan/eng/list/index.html) has *K*_m_ below 50 μM and the use of uric acid below 0.10 mM is sufficient for estimating its *K*_m_. Hence, to test the estimation of *K*_m_, the absorbance of uric acid was measured at 308 nm with the recombinant wildtype uricase of *Bacillus* fastidious A.T.C.C. 29604, but at 293 nm with the uricase from Asahi-Kasei, for the maximal absorbance of uric acid within the measurable range of the spectrophotometer. Initial rates were average velocities when uric acid consumption percentages were below 10% after 40 s since reaction initiation. *K*_m_ was estimated by regression analyses according to Lineweaver-Burk plot with determination coefficients over 0.97.

### Assay of total proteins

Protein quantity in a lysate was measured by the Bradford method [38]. All operations completely followed those described in the publication. Specific activity was calculated with the level of total proteins in a lysate as determined.

### Operation procedure for screening uricase mutants

After purification via two consecutive DEAE-Cellulose chromoatography followed by preparative polyacrylamide gel electrophoresis (PAGE), uricase catalytic capacity was the highest specific activity determined with 75 μM uric acid in the borate buffer at pH 9.2. Four uricase mutants of the bacterial uricase as active homotetramers were utilized and denoted as candidate A, B, C and D with their catalytic capacities in a descent order. Among those four candidates, any two uricases were randomly selected into a pair. For such pairs of uricases, the ratios of their catalytic capacities ranged from about 1.3 to about 4.0. Their sequences and kinetic parameters were provided in Additional file 1 (sheet 1, mutant sequences and summary).

To mimic the procedure of screening, the pET28a vector of each mutant was transformed into *Escherichia* coli BL21 (DE3) cells. From each vector, a total of 30 clones on a selective plate with karnamycin were picked one-by-one; every clone was transferred into 1.0-mL selective medium in a 5.0-ml Ependorf tube for cultivation at 37°C for 3 h. Then, IPTG for final 1.0 mM was added to induce the expression of uricase for 18 h at 16°C. Cells from each tube were harvested by centrifugation and lyzed by sonication treatment in ice-water bath at 150 W for 2.0 min (continuous treatment for 2.5 s at intervals of 2.5 s, Ningbo Xinzhi Biomedicine LTD, Zhejiang, China; http://www.csb17.cn/Photo_Show.asp?InfoId=231, model JY92-II sonics cell lysis equipment); insoluble materials were removed by centrifugation at 10,000 rpm for 10 min again. The untransformed *E*. coli BL21 cells contained no endogenous uricase and thus activities of uricases in cell lysates were directly measured. In each clear lysate, uricase activity after proper dilution was measured in duplicate by monitoring UV absorbance at 293 nm with 75 μM uric acid in 0.20 M sodium borate buffer at pH 9.2. The dilution ratio for each candidate was optimized. Abundance of uricase protein in such lysates was examined by SDS-PAGE followed by staining proteins, and further by PAGE to visualize the active forms of uricases via the staining of activity (the staining reaction produces the insoluble visible chromogen through the coupled peroxidase action on a chromogenic substrate and hydrogen peroxide released by the action of uricase on uric acid) [39].

### Data processing and recognition of a mutant of higher activity

Coefficient of variation (CV) was deduced from standard deviation (SD) and mean, and was compared by *F*-test to examine homoscedasticity. Statistical comparisons and correlation analyses were made with MS Excel 6.0. Normality of data was examined with Shapiro-Wilk test incorporated in SPSS 17.0.

In each pair of two uricases, the one of lower catalytic capacity is taken as the starting material and the other of higher catalytic capacity is designated as the positive candidate. Two methods were used for the recognition of a positive candidate in a pair with its activity measured in a lysate. Firstly, statistical comparison of averages of activity indexes in lysates of two uricases in a pair was used. Secondly, data in each pair were analyzed by receiver-operation-curve (ROC) analysis with SPSS 17.0 to determine the area-under-the-curve (AUC) [40,41]. The response of AUCs to ratios of catalytic capacities of paired uricases was examined. The minimal ratio in the catalytic capacities of a pair of uricases for an AUC of about 1.00 was selected; from ROC of this pair of two uricase, a threshold was selected for the sensitivity of 90% and specificity as close to 90% as possible. Such a threshold was normalized with respect to the mean and SD of the starting material to test its universal applicability to other pairs of uricases.

## Results and discussion

### Verification of the assay of uric acid absorbance for measuring initial rate

For screening mutants, uricase activities are reflected by change rates of uric acid absorbance during initial rate reaction. The interference of HIU with the assay of uricase initial rate by measuring uric acid absorbance depends on extinction coefficient and dynamic levels of HIU. When uric acid consumption percentages are low enough for initial rate reaction, there will be no interference from HIU with the assay of uricase initial rate and thus a linear decrease of uric acid absorbance if HIU levels remain steady, or else, there will be interference from HIU with the assay of uricase initial rate and thus a nonlinear decrease of uric acid absorbance. Steady-state level of HIU and its lag time are determined by its production and decomposition rates. Steady-state of uricase reaction requires a lag time of about 40 s [35,36]. When the consumption of uric acid is within 10%, nonlinear decrease of uric acid absorbance after a lag time of 40 s directly supports that HIU levels do not achieve steady-state. If steady-state of HIU is achieved after so long lagging periods that the consumption percentages of uric acid exceed the limits for initial rate reaction, there will be inaccurate initial rates of uricases and such deviations are more pronounced with uricases of higher *K*_m_. Any factor affecting the production and decomposition rates of HIU including uricase activity, the use of Tris–HCl buffer or borate buffer, and reaction pH, will alter the steady-state levels of HIU and thus its interference with the assay of uricase initial rate by measuring uric acid absorbance [30,31]. On the other hand, the wavelength to quantify uric acid absorbance also alters extinction coefficient of HIU and thus its interference with the assay of uricase initial rates. The absorbance peak of uric acid locates at 293 nm while that of HIU is around 302 nm [30,31]. The bacterial uricase has *K*_m_ over 0.20 mM for uric acid [32,33]; the highest level of uric acid should be about 0.4 mM to estimate its *K*_m_. Only at wavelengths no shorter than 308 nm, absorbance for uric acid at 0.4 mM is below 1.200 and measurable with common spectrophotometers; the interference from HIU with the assay of uricase initial rate by measuring uric acid absorbance should be more manifest at 308 nm than at 293 nm. The bacterial uricase is thus an ideal model to test the existence and solution of the interference from HIU with the assay of uricase initial rate. Moreover, uricase activities will alter the interference from HIU with initial rates and thus *K*_m_ estimated via Lineweaver-Burk plot analysis of initial rates, which violates the facts with common enzymes. Hence, the interference from HIU with the assay of uricase initial rates by uric acid absorbance is examined based on: (a) effects of uricase activities, reaction buffers and pH values on nonlinear decreases of absorbance of uric acid when its consumption percentages are below 10%, (b) effects of wavelengths to quantify uric acid absorbance on nonlinear decreases of absorbance of uric acid when its consumption percentages are below 10%, and (c) the effects of uricase activities on *K*_m_.

**Figure 1:**
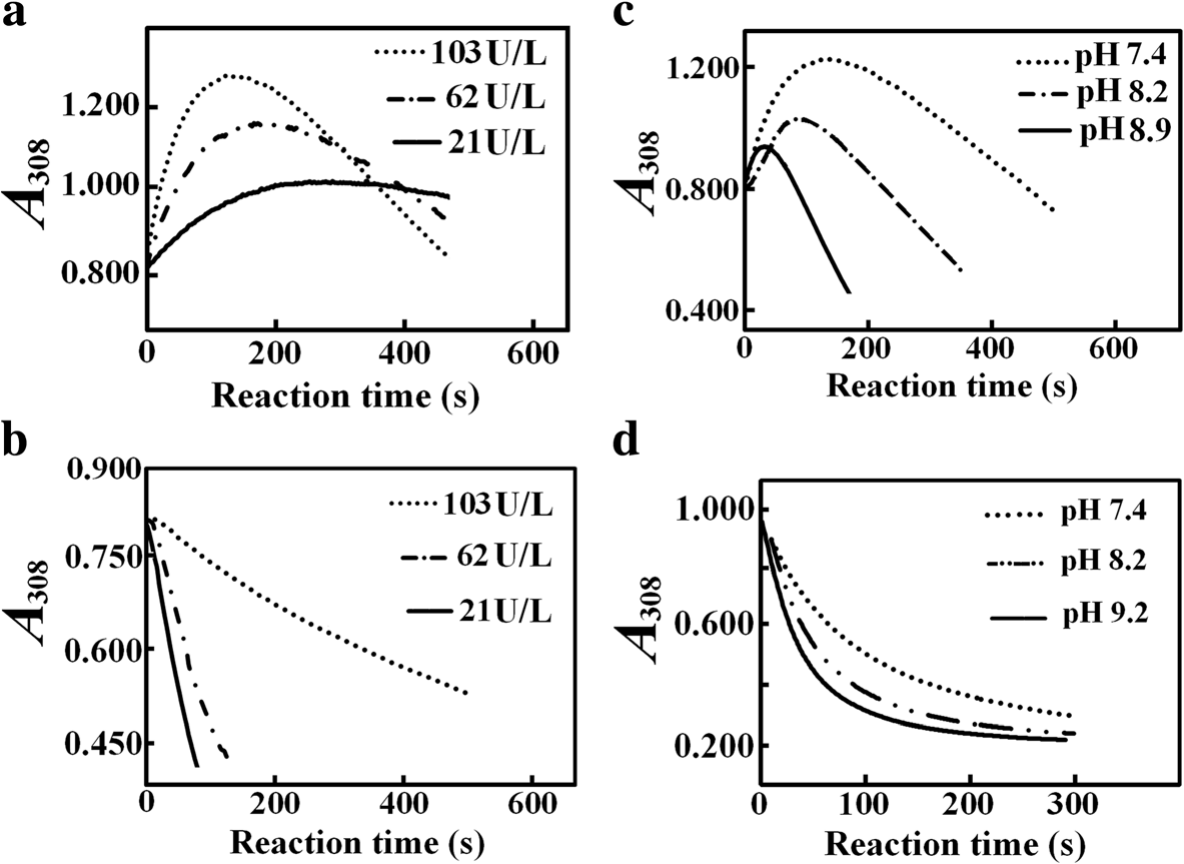
**Interference from HIU with the assay of absorbance at 308 nm after the lag time of 10 s for 0.30 mM uric acid under uricase action at 25°C. a**. Effects of activities on interference in 0.10 M Tris–HCl buffer at pH 7.4 **b**. Effects of activities on interference in 0.20 M borate buffer at pH 7.4 **c**. Effects of reaction pH on interference in 0.10 M Tris–HCl buffer with final uricase activity at 103 U/L **d**. Effects of reaction pH on interference in 0.20 M borate buffer with final uricase activity at 103 U/L.

**Figure 2:**
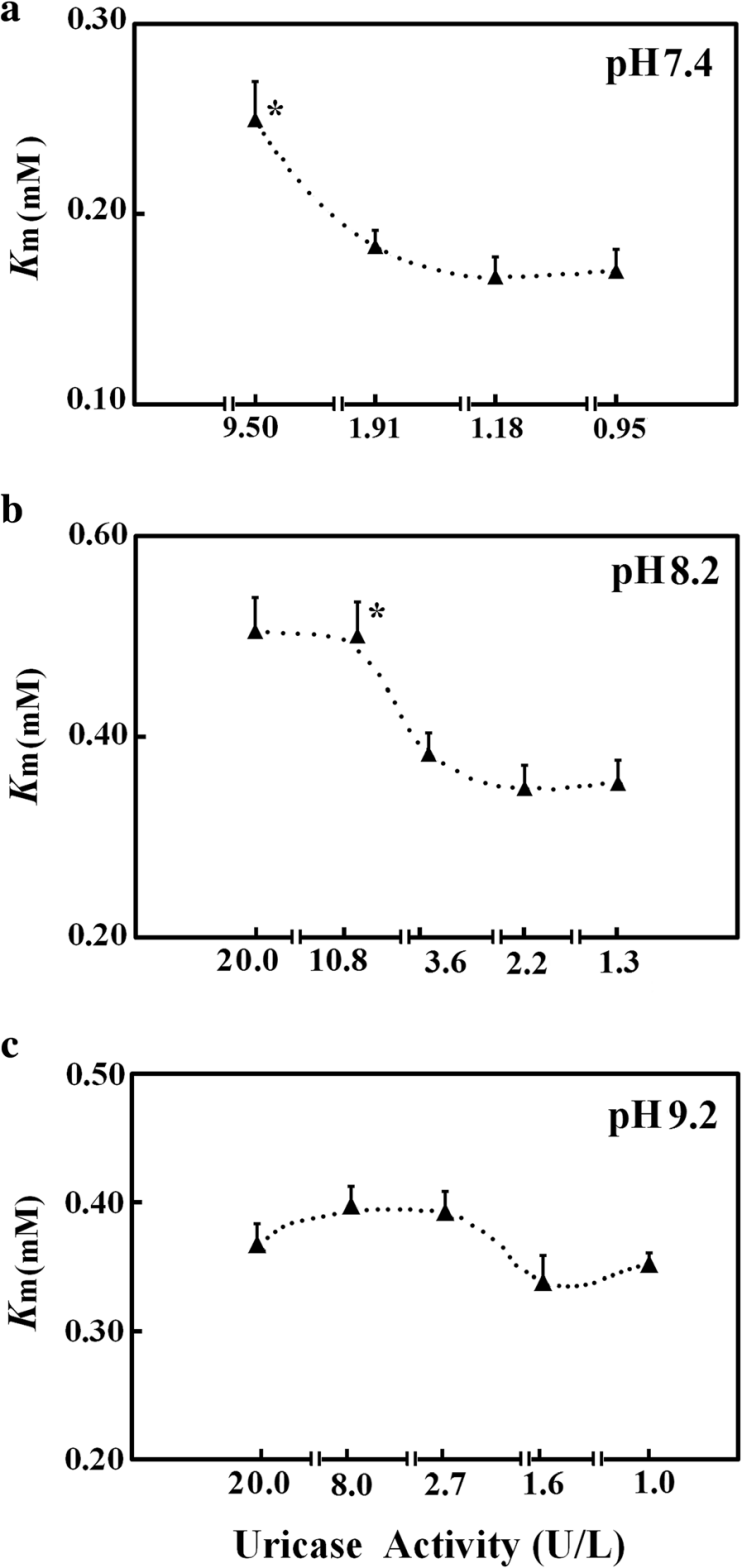
**Effects of uricase activities on *K***_**m **_**estimated by measuring absorbance at 308 nm in 0.20 M borate buffers at 25°C.** Final uric acid concentrations ranged from 0.10 to 0.50 mM at pH 9.2 or 8.2, and from 0.070 to 0.33 mM at pH 7.4. Results were from at least three independent assays with CVs below 12%. Uricase activities were those calibrated with 0.075 mM uric acid at the indicated reaction pH. ^*^ indicated *P* < 0.05 *versus* that at lower uricase activities by Student *t*-test.

In Tris–HCl buffer at 7.4, the interference from HIU with the absorbance of 0.30 mM uric acid at 308 nm was detected as a continuous increase rather than decrease in absorbance till a peak at about 100 s since reaction initiation, even the consumption of uric acid was within 10%; the use of a higher uricase activity produced a larger increase in absorbance at 308 nm after 100 s since reaction initiation (Figure 1). These results supported that HIU levels do not achieve steady-state within 100 s since reaction initiation. The interference from HIU with uric acid absorbance in Tris–HCl buffers was alleviated by the increase in reaction pH or the use of magnesium ion (Additional file 2: Figure S1, S2). After reaction initiation at 103 U/L uricase, there were no detectable increases in absorbance at 293 nm but tiny increases in absorbance at 308 nm with 0.12 mM uric acid in Tris–HCl buffer at pH 7.4 (Additional file 2: Figure S3), and there were also no increases in absorbance at 308 nm with 0.30 mM uric acid in 0.20 M sodium borate buffers at pH 7.4 (Figure 1); those results supported the interference from HIU with uric acid absorbance is weaker at 293 nm than at 308 nm and is even negligible in borate buffers at both wavelengths. At 400 U/L uricase, uric acid concentration after the lag time of 20 s in the borate buffer at pH 7.4 was smaller than 0.27 mM, but there were still no increases in absorbance at 308 nm. No increases in absorbance at 308 nm during uricase reaction were detected in borate buffers at higher pH. On the other hand, when activities of the recombinant uricase were decreasing, *K*_m_ by measuring absorbance at 308 nm in borate buffers decreased to stable values (Figure 2). The use of a higher reaction pH enabled the estimation of such a stable *K*_m_ at higher uricase activities. For Asahi-Kasei uricase with *K*_m_ of about 30 μM, the highest uric acid levels were below 0.10 mM so that uric acid absorbance can be measured at 293 nm in borate buffers to determine initial rates for estimating its *K*_m_; there were no changes of *K*_m_ over a wide range of uricase activities. Moreover, by measuring absorbance at 293 nm with 0.075 mM uric acid in borate buffer at pH 9.2, initial rates linearly responded to uricase quantities ranging from 0.6 to about 12 U/L when absorbance was monitored at 5-s intervals. Repetitive assays for uricase activities from 3.0 U/L to 12 U/L with a pooled lysate showed CVs below 5.5% (*n* = 11).

Taken together, the assay of absorbance of uric acid at 293 nm in alkaline borate buffers is reliable for measuring uricase initial rates within a reasonable range.

### Recognition of a mutant of higher activity

To recognize positive candidates in a library, a decision method should have sensitivity and specificity as high as possible. One-fold or 100% improvement of catalytic capacity of a mutant already makes sense and the recognition of such a mutant is a challenge. Hence, four uricases with ratios of catalytic capacities from about 1.3-fold to about 4-fold were employed as models to test the potential solutions to the challenge.

**Figure 3:**
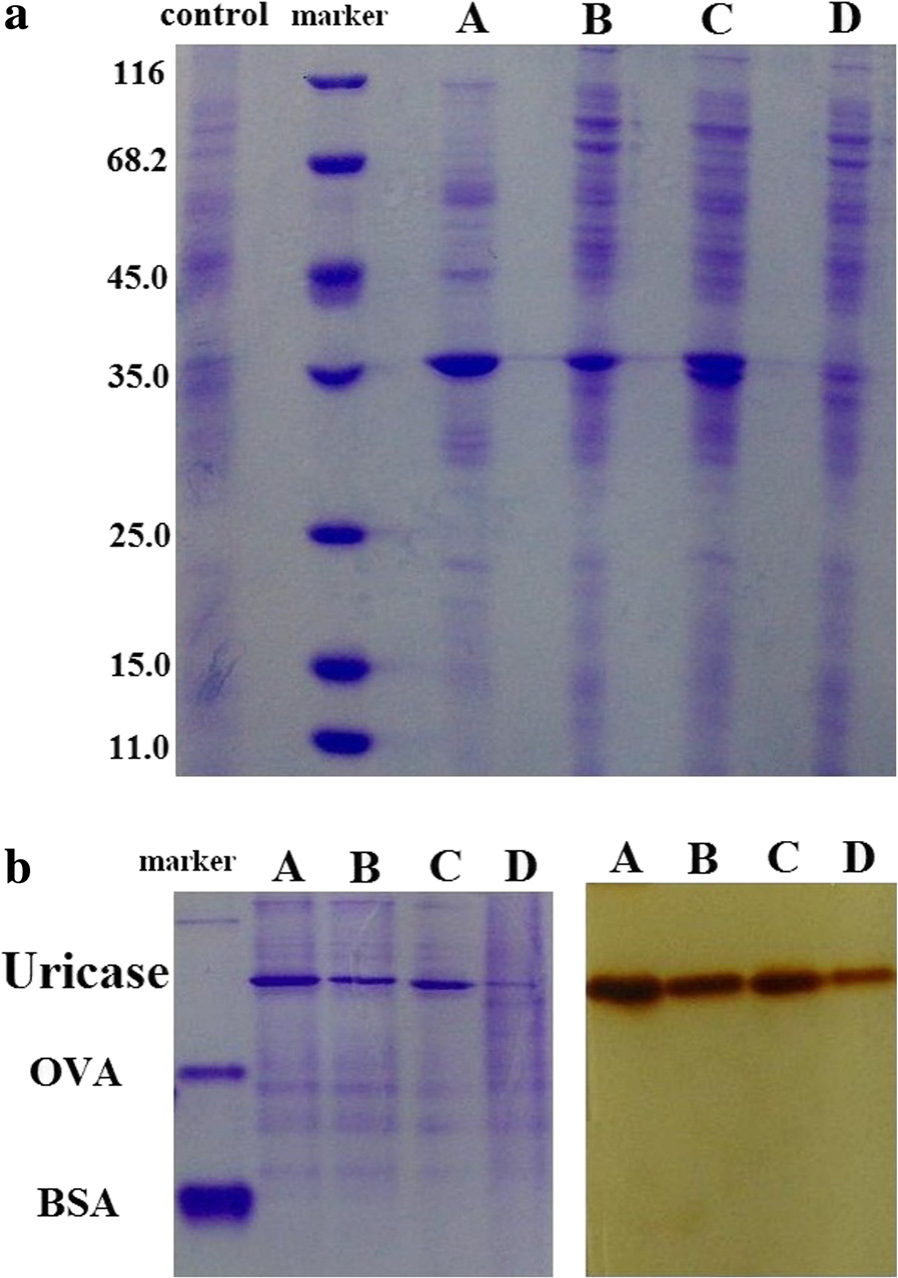
**PAGE analyses of proteins in lysates of four uricase mutants.** (**a**) SDS-PAGE and staining of protein bands. (**b**) PAGE followed by the staining of protein bands (left side) and uricase activity (right side).

The abundance of uricase proteins in lysates was examined at first. With the same quantities of total proteins for analyses, band density of four uricases was different after SDS-PAGE followed by staining of protein bands, or after PAGE followed by staining of uricase activity (Figure 3). The averages of total proteins in lysates of four uricase mutants had no statistical differences, but their activity concentrations displayed positive associations with the levels of total proteins in lysates; those results supported the concern on negative impacts of variations of the levels of mutant proteins in lysates on the recognition of positive candidates. Activity concentration of uricases in lysates basically followed normal distribution, but specific activities of most candidates displayed non-normal distribution (Table 1). Candidate D had the lowest abundance and the lowest specific activity. The ratios of specific activities among candidate A, B and C showed no significant deviations from those of catalytic capacities, but the ratios of specific activities of candidate A, B and C to that of candidate D were more than twice of those of catalytic capacities, respectively (Table 2). Such large deviations in ratios of specific activities of the three uricase mutants to candidate D from those of their catalytic capacities should be associated with the lower abundance of candidate D in its lysate. Hence, the variations in the abundance of mutant proteins in lysates should be considered carefully.

**Table 1:** **Uricase activities at 0.075 mM uric acid and protein levels in lysates (*n*= 30)**

**Candidates/parameters**	**A**	**B**	**C**	**D**
Total proteins by the Bradford assay	0.63	0.66	0.65	0.66
	(0.09)	(0.10)	(0.15)	(0.09)
Activity concentration	0.30	0.28	0.18	0.033
	(0.07)	(0.06)	(0.05)	(0.004)
*P* for normality of AC by shapiro-Wilk test	0.104	0.182	0.439	0.282
Correlation coefficient (*R*)	>0.63	>0.81	>0.84	>0.44
Specific activity	0.49	0.43	0.28	0.050
	(0.09)	(0.05)	(0.05)	(0.006)
*P* for normality of SA by Shapiro-Wilk test	<0.001	0.310	0.972	0.031

Numbers in parentheses were SD. Correlation coefficient was that for the association of activity concentrations with levels of total proteins. *R* > 0.361 and > 0.464 indicated *P* < 0.05 and *P* < 0.01 for the association of activity concentrations with proteins levels, respectively. SA: specific activity; AC: activity concentration.

**Table 2:** **ROC analysis of activities in lysates (*n*= 30)**

**Candidate pairs**	**Ratios after purification**	**Ratio by AC**	**AUC by AC**	**Ratio by SA**	**AUC by SA**
A-B	1.37	1.07	0.559	1.19	0.799
B-C	1.58	1.54	0.895	1.56	0.985
A-C	2.15	1.67	0.921	1.82	1.000
C-D	1.90	5.45	1.000	5.60	1.000
B-D	3.00	8.48	1.000	8.60	1.000
A-D	4.10	9.09	1.000	9.92	1.000

Based on error propagation, the experimental ratios in pairs had CVs about 20%. SA: specific activity; AC: activity concentration.

To reveal the differences between the comparison of activity concentration and specific activity for recognizing positive candidates, ROC analyses were performed [40,41]. Clearly, the smaller differences in catalytic capacities of paired uricases led to smaller AUCs by comparing either activity concentration or specific activity (Table 2). When ratios of catalytic capacities of a pair of uricases were over 3.0, the comparison of either activity index consistently yielded AUCs of 1.000. For uricase pairs with ratios of their catalytic capacities below 2.0, the comparison of specific activity gave higher AUCs than the comparison of activity concentration (Table 2). Hence, for screening uricase mutants, the comparison of specific activity calculated from the levels of total proteins in lysates is favorable for recognizing positive candidates of small improvement of activity.

**Figure 4:**
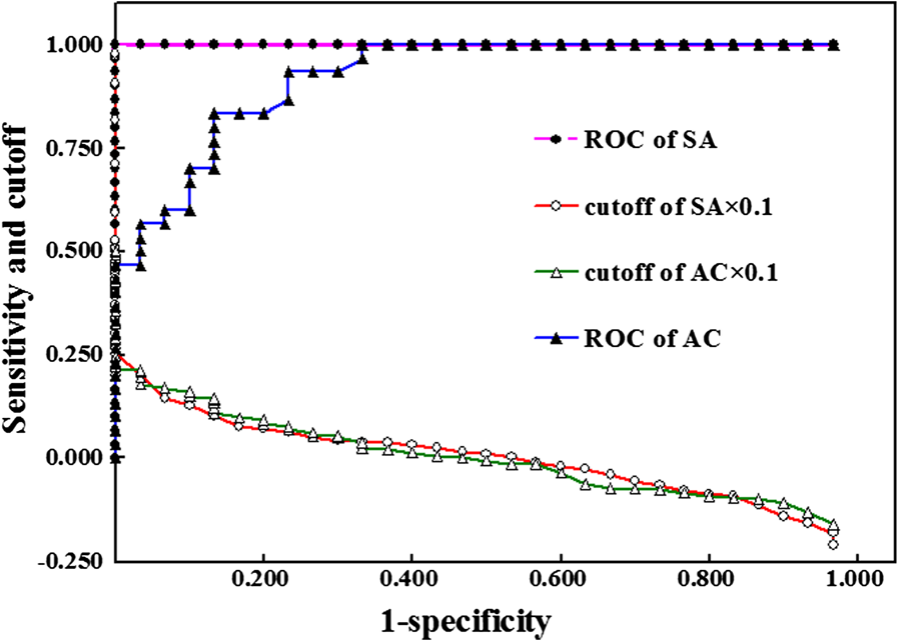
**ROC analysis of the pair of uricase with ratio of about 1.80 for their catalytic capacities and associations of normalized cutoffs (×0.1) with 1-specificity.** SA: specific activity; AC: activity concentration

By the comparison of specific activity to recognize a positive candidate, a practical threshold is required. The ratio of specific activity between candidate A and C was over 1.7 with either crude enzymes or purified enzymes (Additional file 1: sheet 1: mutant sequences and summary), and their activity concentrations and specific activities exhibited consistent CVs (Table 1). The practical threshold for the recognition of a positive candidate was investigated with this pair of uricases. For ROC analysis of the recognition of candidate A as a positive candidate against candidate C, the deviations of the preset cutoffs from the mean of the starting material were divided by the SD of the starting material for plotting against 1-specificty to observe the association of decision sensitivity with the preset cutoffs (Figure 4). For no less than 90% specificity to recognize the positive candidate in this uricase pair, the mean of specific activity plus 1.4-fold of the SD of the starting material can be a practical threshold (Additional file 1: sheet 2: ROC analysis of activity). By using such a threshold to other uricase pairs, the comparison of specific activity always gave higher sensitivity to identify positive candidates than the comparison of activity concentration. Hence, the comparison of specific activity calculated from the levels of total proteins against a suitable threshold is more favorable for recognizing positive candidates with small improvement of catalytic capacity.

## Conclusion

For screening uricase mutants, the assay of UV absorbance of uric acid at 293 nm is effective to measure uricase activities within a reasonable range in alkaline borate buffers. To recognize a mutant of small improvement of catalytic capacity, specific activity calculated from total proteins in a lysate is an index better than activity concentration; a practical threshold for comparison to recognize a positive candidate can be the mean plus 1.4-fold SD of specific activities of the starting material.

## Abbreviations

AUC: Area-under-the-curve; BSA: Beef serum albumin; HIU: 5-hydroisourate; IPTG: Isopropyl-β-*D*-thiogalactoside; Km: Michaelis-Menten constant; OVA: Ovalumin; ROC: Receiver-operation-curve; Tris: Tri-(hydroxylmethyl)-aminomethane.

## Competing interest

The authors declared no competing interest.

## Authors’ contributions

JF, HL, AG, LF, GL, JP and YL performed the experiments; FL, JF, HL and XY analyzed experimental data; XY and FL wrote the manuscript; FL and XY conceived the idea. All authors read and approved the final manuscript.

## Supplementary Material

Additional file 1 **The following additional data are available with the online version of this paper.** Sheet 1: mutant sequences and summary; Sheet 2: ROC analysis of activity. Click here for file

Additional file 2 **The following additional data are available with the online version of this paper.Figure S1.** Effects of magnesium ion on the interference from HIU in Tris–HCl buffer at pH 7.4; **Figure S2.** Effects of magnesium ion on the interference from HIU in Tris–HCl buffer at pH 8.9; **Figure S3.** Interference from HIU with *A*_293_ and *A*_308_ for uric acid in Tris–HCl buffer at pH 7.4. Click here for file
