# Supplementary material for: Comparison of activity indexes for recognizing enzyme mutants of higher activity with uricase as model
Source: Chem Cent J. 2013 Apr 17;7:69. doi: 10.1186/1752-153X-7-69 (PMC3637054; doi:10.1186/1752-153X-7-69)
Supplement: Additional file 2 — The following additional data are available with the online version of this paper.Figure S1. Effects of magnesium ion on the interference from HIU in Tris–HCl buffer at pH 7.4; Figure S2. Effects of magnesium ion on the interference from HIU in Tris–HCl buffer at pH 8.9; Figure S3. Interference from HIU with A293 and A308 for uric acid in Tris–HCl buffer at pH 7.4. [file 1752-153X-7-69-S2.pdf]

Figure S1. Effects of magnesium ion on the interference from HIU in Tris-HCl buffer at pH 7.4

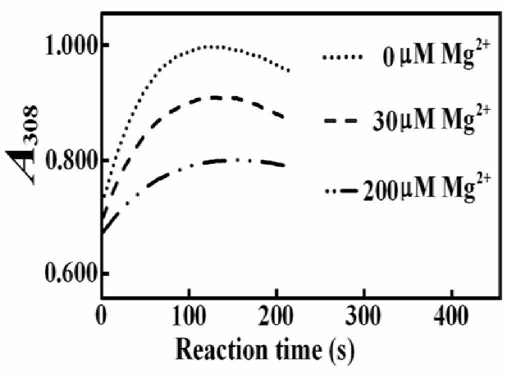

Final 103 U/L uricase was used in 0.10 mol/L Tris-HCl buffer at pH 7.4 containing 0.30 mmol/L uric acid.

Figure S2. Effects of magnesium ion on the interference from HIU in Tris-HCl buffer at pH 8.9

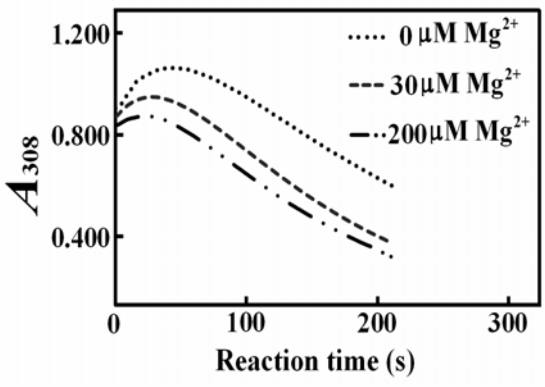

Final 103 U/L uricase was used in 0.10 mol/L Tris-HCl buffer at pH 7.4 containing 0.30 mmol/L uric acid.

Figure S3. Interference from HIU with A_{293} and A_{308} for uric acid in Tris-HCl buffer at pH 7.4

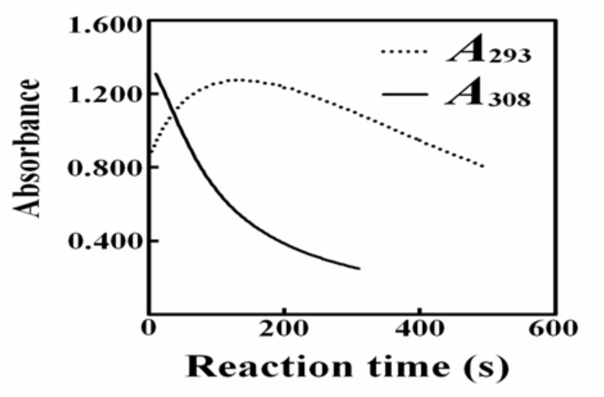

Uricase activities were 103 U/L. Final 0.30 mmol/L uric acid was used to measure *A*308, and 0.12 mmol/L uric acid was used to measure A_{293}.
